# Aboveground and Belowground Plant Traits Explain Latitudinal Patterns in Topsoil Fungal Communities From Tropical to Cold Temperate Forests

**DOI:** 10.3389/fmicb.2021.633751

**Published:** 2021-06-10

**Authors:** Jialing Teng, Jing Tian, Romain Barnard, Guirui Yu, Yakov Kuzyakov, Jizhong Zhou

**Affiliations:** ^1^Key Laboratory of Ecosystem Network Observation and Modeling, Institute of Geographic Sciences and Natural Resources Research, Chinese Academy of Sciences, Beijing, China; ^2^College of Resources and Environment, University of Chinese Academy of Sciences, Beijing, China; ^3^Key Laboratory of Plant-Soil Interactions, Ministry of Education, College of Resources and Environmental Sciences, China Agricultural University, Beijing, China; ^4^Agroécologie, AgroSup Dijon, INRA, Univ. Bourgogne, Univ. Bourgogne Franche Comté, Dijon, France; ^5^Department of Soil Science of Temperate Ecosystems, University of Göttingen, Göttingen, Germany; ^6^Faculty of Life Science and Technology, Central South University of Forestry and Technology, Changsha, China; ^7^Department of Microbiology and Plant Biology, School of Civil Engineering and Environmental Sciences, Institute for Environmental Genomics, University of Oklahoma, Norman, OK, United States; ^8^Earth and Environmental Sciences, Lawrence Berkeley National Laboratory, Berkeley, CA, United States

**Keywords:** community composition, forest ecosystems, leaf traits, root traits, soil fungi

## Abstract

Soil fungi predominate the forest topsoil microbial biomass and participate in biogeochemical cycling as decomposers, symbionts, and pathogens. They are intimately associated with plants but their interactions with aboveground and belowground plant traits are unclear. Here, we evaluated soil fungal communities and their relationships with leaf and root traits in nine forest ecosystems ranging from tropical to cold temperate along a 3,700-km transect in eastern China. Basidiomycota was the most abundant phylum, followed by Ascomycota, Zygomycota, Glomeromycota, and Chytridiomycota. There was no latitudinal trend in total, saprotrophic, and pathotrophic fungal richness. However, ectomycorrhizal fungal abundance and richness increased with latitude significantly and reached maxima in temperate forests. Saprotrophic and pathotrophic fungi were most abundant in tropical and subtropical forests and their abundance decreased with latitude. Spatial and climatic factors, soil properties, and plant traits collectively explained 45% of the variance in soil fungal richness. Specific root length and root biomass had the greatest direct effects on total fungal richness. Specific root length was the key determinant of saprotrophic and pathotrophic fungal richness while root phosphorus content was the main biotic factor determining ectomycorrhizal fungal richness. In contrast, spatial and climatic features, soil properties, total leaf nitrogen and phosphorus, specific root length, and root biomass collectively explained >60% of the variance in fungal community composition. Soil fungal richness and composition are strongly controlled by both aboveground and belowground plant traits. The findings of this study provide new evidence that plant traits predict soil fungal diversity distribution at the continental scale.

## Introduction

Interactions between plants and soil fungi are vital to the normal functioning of plant-soil systems such as forest ecosystems. Fungal life cycles are intimately linked to those of land plants (Barberan et al., 2015). In forest soils, fungi participate in crucial ecosystem processes as decomposers, symbionts, or phytopathogens (Tedersoo et al., 2014; Schimann et al., 2017). Saprotrophic fungi decompose complex organic matter and render carbon and other nutrients bioavailable to plants (Cairney and Meharg, 2002; Goldmann et al., 2015). Ectomycorrhizal (EcM) fungi form mutualistic relationships with host plants. They infect roots and promote plant nutrient and water uptake (Nehls, 2008; Goldmann et al., 2015). Plant pathogenic fungi and mutualists worked together in driving spatiotemporal patterns of plants (Chen et al., 2019). Soil fungi mediate soil structure development at various spatial scales and control water and nutrient flow and root growth and distribution (Ritz and Young, 2004). Their detritus substantially adds to forest soil organic carbon stocks (Clemmensen et al., 2013). Fungi are ubiquitous and play pivotal roles in ecosystem services. Nevertheless, the factors influencing their global-scale diversity and biogeography remain unclear (Cox et al., 2016).

Recent studies documented numerous biotic and abiotic factors that influence soil fungal diversity and community structure across wide spatial scales and in response to limited dispersal and geographic isolation (Cox et al., 2016), climate (temperature and precipitation; Tedersoo et al., 2014; Zhou et al., 2016), soil properties (texture, bulk density, pH, nutrient availability, and organic matter content) (Leifheit et al., 2014; Guo et al., 2019), and plant diversity (Peay et al., 2013; Cline et al., 2018). Plants affect soil fungi via organic matter inputs such as litter and rhizodeposits (Wardle et al., 2004; Bardgett and van der Putten, 2014). They also alter local soil abiotic conditions such as soil structure, moisture, pH, and oxidation-reduction potential (Waring et al., 2015). Therefore, plant diversity influences the quantity and composition of plant inputs and could, by extension, also affect soil fungal diversity. However, plant and fungal communities strongly depend upon climatic and edaphic variables. Thus, the relationships between plant species and soil fungal diversity may be contingent upon the spatial scale and the type of diversity. Plant-fungal α- and β-diversity relationships occur at the local and regional scales (Cline et al., 2018). In contrast, only global-scale β-diversity but no α-diversity relationships have been detected (Tedersoo et al., 2014; Prober et al., 2015). These findings suggest that plant diversity may be not a reliable indicator of the interactions between plants and soil fungi. However, there is growing consensus that plant functional traits determine diversity effects (Cardinale et al., 2012; Bardgett and van der Putten, 2014; Li et al., 2017). This postulate is consistent with the fact that natural ecosystems are inherently complex.

Plant functional traits are measurable and associated with productivity or adaptation to the environment (Violle et al., 2007; He et al., 2019). Community-weighted means (CWM) of plant functional traits represent the dominant community trait values and reflect “optimal” trait strategies under local environmental conditions (Díaz et al., 2007; Garnier and Navas, 2012). The latter parameters are related to community- and landscape-scale soil biological communities and ecosystem processes (de Vries et al., 2012; Pei et al., 2016; Buzzard et al., 2019; He et al., 2019). Combinations of plant traits indicate the quantity and quality of litter input affecting the soil microbial community (Wardle et al., 2004; Orwin et al., 2010; Reich, 2014). It is necessary to determine the extent to which plant traits explain variations in soil processes. However, few studies have attempted to evaluate the connections between plant functional traits such as leaf and root morphology and nutrient content and soil fungal community structure across broad environmental gradients (Chua and Potts, 2018; Buzzard et al., 2019; Van der Plas et al., 2020; Wang et al., 2020).

The leaves that constitute the bulk of forest litter determine litterfall quantity and quality. Variations in leaf traits directly and indirectly affect soil-substrate quality by influencing the soil microbial community and biogeochemical processes (Buzzard et al., 2019). CWM of leaf traits such as specific area, dry matter content, and nitrogen concentration explain most of the variation in litter decomposition rate (Quested et al., 2007; Fortunel et al., 2009; Eichenberg et al., 2015), soil nutrient cycles, and belowground processes (Laughlin, 2011; de Vries et al., 2012; Makkonen et al., 2012; Freschet and Cornelissen, 2013). Recent studies confirmed the relationships among leaf N concentration, biomass, specific area, and soil fungal community (de Vries et al., 2012; Pei et al., 2016; De Long et al., 2019). Unlike leaves, roots make contact with the soil and directly participate in soil ecological processes (Bardgett et al., 2014; Moreau et al., 2015; de Vries et al., 2016; Bardgett, 2017). Trees allocate substantial amounts of their recently assimilated carbon (C) to their roots. Fine root decomposition and root exudates are major C contributors to forest soil microbial communities and regulate microbial metabolism (Pollierer et al., 2007; Hogberg et al., 2008; Freschet and Cornelissen, 2013; Ponge, 2013). The priming effect of easily metabolizable C inputs from roots control soil organic matter decomposition and nutrient release (Fontaine et al., 2007; Cheng, 2009; Kuzyakov, 2010). Root traits are closely associated with various soil processes (Bardgett et al., 2014; Faucon et al., 2017; Yang et al., 2021). The chemical traits of fine roots are the best indicators of community-level root decomposition and root exudates (Prieto et al., 2016). Fine root decomposition is negatively correlated with lignin and positively correlated with root nitrogen (N) and phosphorus (P) levels (See et al., 2019). The root C:N ratio explains root exudate quality and quantity (Vale et al., 2005; Legay et al., 2014). Fine roots with large specific root lengths (SRL) are positively correlated with fungal: bacterial (F: B) ratios (Legay et al., 2014).

Clarification of the linkages between plant functional traits and soil microbial communities could be capable of identifying the most relevant plant drivers in the prediction of ecosystem process changes based on plant community responses (Lavorel and Garnier, 2002; Funk et al., 2017). However, previous studies focused primarily on the effects of plant traits on aboveground plant litter decomposition. In contrast, there is relatively little information about the relationships between the combined attributes of plant shoots and roots and those of soil fungi (Legay et al., 2014; Yang et al., 2020). Hence, the present study explored the relationships between soil fungal diversity and plant traits across the wide latitudinal and temperature gradients of the North-South Transect of Eastern China (NSTEC). The NSTEC includes forest types ranging from cold temperate to tropical and contains experimental sites varying in climate, edaphic, and plant factors. Here, we hypothesize that (a) changing plant functional traits directly affect soil fungal diversity and community composition from tropical to cold temperate forests, and (b) root traits more strongly and directly influence soil fungal community compositions than leaf traits.

## Materials and Methods

### Study Area and Plant and Soil Sampling

Nine forest sites in protected national nature reserves along the NSTEC were selected to represent typical vegetation and soil characteristics of (1) cold temperate coniferous forests (Huzhong, HZ), (2,3) temperate conifer broadleaf mixed forests (Liangshui, LS; Changbai, CB), (4,5) warm temperate deciduous broadleaf forests (Dongling, DL; Taiyue, TY), (6) subtropical deciduous evergreen mixed forest (Shennong, SN); (7,8) subtropical evergreen broadleaf forests (Jiulian, JL; Dinghu, DH), and (9) tropical monsoon forests (Jianfeng, JF). The forest sites spanned broad latitudinal (18°44′–51°46′N, 128°53′–108°51′E; Supplementary Figure 1) and climatic (Supplementary Table 1) gradients. The mean annual temperatures (MAT) and mean annual precipitation (MAP) levels ranged from −3.67 to 23.15°C and from 473 to 2,266 mm, respectively. Climatic variables including MAT and MAP were acquired from the CERN meteorological database.

In July 2013, four experimental plots (30 m × 40 m) were established at each site. The plant species in each plot, including woody and non-woody species, were surveyed and species richness was used to describe plant diversity (PD) (Supplementary Table 1). The leaves and roots of dominant and common plant species were collected according to a unified protocol (Zhao et al., 2018). In each plot, 20 surface soil cores (0–10 cm depth) were collected and pooled (see Tian et al., 2018) for details of the soil sampling procedure). Soil samples from each plot were sieved through a 2-mm mesh and blended into a single representative sample per plot. Visible roots and organic debris were carefully removed. A subsample of each soil sample was placed in a 50-mL centrifuge tube, stored in an icebox, transported to the laboratory, and maintained at −80°C for subsequent soil DNA extraction. Physicochemical analyses were performed on the remaining soil.

### Physicochemical Analysis of Plants and Soils

As described by Wang et al. (2018) and Zhao et al. (2018), we collected sun-exposed and mature leaves (leaf blades for trees) and fine roots (diameter < 2 mm) from between five and ten individuals of each plant species at each site. The leaf and fine root samples were carefully cleaned and oven-dried at 60°C. All samples were ground to fine powder, using a ball mill (MM400 Ball Mill, Retsch, Germany) and an agate mortar grinder (RM200, Retsch, Haan, Germany), for element analysis. The root length was obtained by analyzing the scanned root samples with WinRHIZO 2009 (Regent Instruments, Quebec, Canada). SRL was calculated as root dry mass divided by root length. CWM of leaf and root C, N, and P (leaf C, leaf N, leaf P, root C, root N, and root P) were determined based on their respective characteristic values for each species and relative species abundance (Supplementary Table 1; Zhao et al., 2018). Root biomass and specific root length (SRL) were obtained from Wang et al. (2018) (Supplementary Table 1).

The pH of 1:2.5 w/v soil suspensions were measured with a pH meter. Total soil organic carbon (SOC) and total nitrogen (TN) were determined with an elemental analyzer (Vario EL III; Elementar Analysensysteme GmbH, Langenselbold, Germany). Soil samples were digested with H_2_SO4-H_2_O_2_-HF and the soil total P concentration (mg kg^–1^) was determined by the ammonium molybdate method in an AutoAnalyzer 3 continuous-flow analyzer (Bran + Luebbe GmbH, Norderstedt, Germany).

### DNA Extraction and Amplicon Sequencing

Soil DNA was extracted with a PowerSoil kit (MoBio Laboratories, Carlsbad, CA, United States) according to the manufacturer’s instructions. Purified DNA quality was evaluated by calculating the DNA absorbance ratios at 260/280 nm and 260/230 nm in a NanoDrop ND-1000 spectrophotometer (NanoDrop Technologies Inc., Wilmington, DE, United States).

The primers ITS1F (5′-CTTGGTCATTTAGAGGAAGTAA-3′) and 2043R (5′-GCTGCGTTCTTCATCGATGC-3′) were used for amplification. After PCR and purification, a DNA library was constructed and run on a MiSeq Illumina platform at Majorbio Bio-Pharm Technology Co., Ltd., Shanghai, China.

Sequencing data were analyzed with Trimmomatic v.0.32 (Bolger et al., 2014) and FLASH v.1.2.4 (Magoč and Salzberg, 2011) software. The soil fungal richness and community composition were calculated and analyzed with Mothur v. 1.30.11 (Schloss et al., 2009). The number of operational taxonomic units (OTUs) was obtained with Usearch v. 7.1 (Edgar, 2010) using the furthest-neighbor algorithm. Each OTU had > 97% sequence similarity. A randomly selected subset of 32,133 sequences per sample was used in the subsequent analysis.

Functional fungal guilds were assigned with FUNGuild (Nguyen et al., 2016). OTUs were assigned to ectomycorrhizal (EcM) mutualists, saprotrophs, and plant pathotrophs. Fungi representing < 1% of the OTUs were not considered.

### Statistical Analysis

The number of OTUs indicates phylotype richness. Shapiro-Wilk test and Bartlett’s test were used to check the normal distributions and homoscedasticity of variances of the richness. Depending on the normal distribution and homogeneity of variance, one-way ANOVA and Tukey’s *post hoc* comparisons were used to evaluate the significant differences among sampling sites. Environmental variables were logarithmic transformed and then we used Pearson correlation to evaluate the correlations between fungal richness and environmental variables. The best ordinary least squares (OLS) multiple regression models for fungal richness and the environmental factors were selected. Corrected Akaike Information Criterion (AICc) was used to identify the best OLS model which penalizes overfitting. For this purpose, the MASS 7.3–51.6 (Venables and Ripley, 2010) package in R 3.6.3 (R Core Team, Vienna, Austria) was used. The variance inflation factor (VIF) was calculated to remove strongly multicollinear variables and select variables suitable for OLS multiple regression models. For this purpose, the CAR 2.1–2 package (Weisberg and Fox, 2011) in R 3.6.3 (R Core Team, Vienna, Austria) was used. Random forest analyses identified the most important predictors of soil fungal richness and community composition. For this purpose, the rfPermute 2.1.81 package (Archer and Kimes, 2008) in R 3.6.3 was used.

Non-metric multidimensional scaling (NMDS) based on Bray-Curtis distance at the OTU level assessed the changes in fungal community composition. Analysis of similarities (ANOSIM) was performed to evaluate differences in fungal composition at the OTU level within sample pairs. For this purpose, the vegan 2.5-6 package (Jari Oksanen et al., 2019) in R 3.6.3 was used.

A partial Mantel test evaluated the connections between the fungal community structure and the measured environmental variables. A canonical correspondence analysis-based variation partitioning analysis (VPA) was implemented to establish the importance of spatial, climatic, edaphic, and plant factors in shaping the fungal community structure. Principal coordinates of neighbor matrices (PCNM) vectors with significant positive spatial autocorrelations were taken as proxies for spatial variables (Borcard et al., 2011). Partial Mantel and VPA were performed in the vegan 2.5-6 package (Jari Oksanen et al., 2019) in R 3.6.3. Matrices of the pairwise taxonomic distances between fungal communities were plotted using the Bray-Curtis dissimilarity index. The Euclidean distances between plant traits were constructed in the vegan 2.5-6 package (Jari Oksanen et al., 2019) in R 3.6.3.

Partial least-squares path models (PLS-PMs) were used to identify the direct and indirect influences of spatial and climatic factors, soil properties, and plant traits on soil fungal richness and community composition (Chin and Dibbern, 2010; Leguina, 2015; De Long et al., 2019). The aforementioned parameters represented latent variables in accordance with their relative importance as indicated by the Random Forest and Mantel tests. The leaf and root traits in the model were CWMs. The models were plotted with the plspm 0.4.9 package (Sanchez, 2012) in R 3.6.3.

## Results

### Taxon and Phylotype Distribution

We obtained 1,156,788 quality sequences for all soil samples. We identified an average of 32,133 sequences per sample and grouped them into 5,266 operational taxonomic units (OTUs) at the 97% similarity level.

**FIGURE 1:**
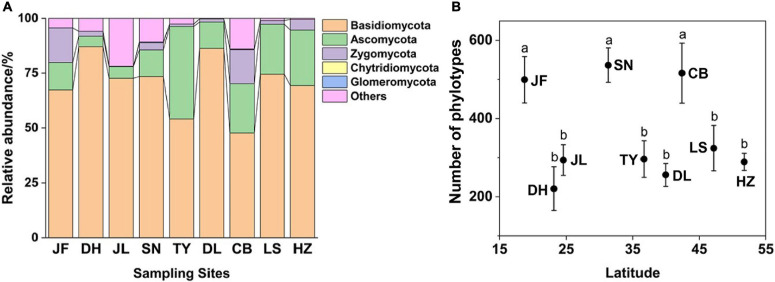
Relative abundance of dominant fungal phyla **(A)** and phylotype richness **(B)** of nine forest ecosystems along north-south transect in eastern China. The sampling sites from south to north in order are JF, DH, JL, SN, TY, DL, CB, LS, and HZ. Different lowercase letters indicate significant differences among means (*p* < 0.05). Error bars represent standard error of the mean.

At the phylum level, Basidiomycota were the most abundant followed by Ascomycota, Zygomycota, Glomeromycota, and Chytridiomycota (Figure 1A). The dominant classes were Agaricomycetes, Leotiomycetes, and Zygomycota_incertae_sedis (relative abundance > 5%), and they accounted for > 60% of all fungal sequences (Supplementary Figure 2). Eurotiomycetes, Microbotryomycetes, Sordariomycetes, Dothideomycetes, Pezizomycetes, Wallemiomycetes, and Archaeorhizomycetes were detected at relatively low abundances in nearly all of the soils analyzed (Supplementary Figure 2).

### Fungal α-Diversity and Community Structure

Soil fungal richness significantly varied across the nine forest ecosystems and displayed no clear latitudinal trend. Fungal richness was greatest in SN, CB, and JF (*p* < 0.05; Figure 1B). At the phylum level, only the relative abundance of Ascomycota increased with latitude (*r* = 0.446; *p* < 0.01; Supplementary Figure 3). The richness (*R*^2^ = 0.13; *p* < 0.05) and relative abundance (*R*^2^ = 0.14; *p* < 0.05) of EcM fungi increased with latitude (Supplementary Figure 4). The relative abundance of saprotrophic (*R*^2^ = 0.17, *p* < 0.05) and pathotrophic (*R*^2^ = 0.04, *p* < 0.05) fungi decreased with latitude (Supplementary Figure 4), while their richness showed no obvious trend with latitude.

**FIGURE 2:**
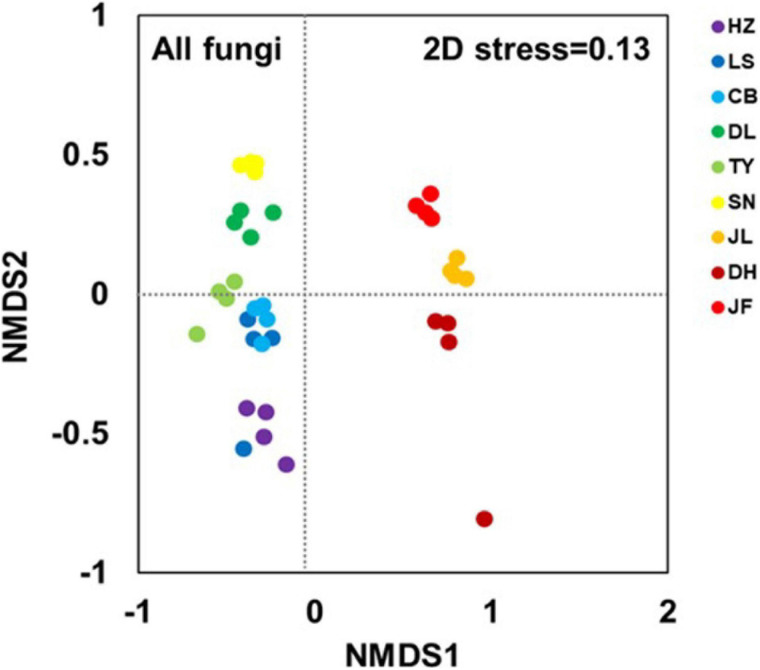
Non-metric multidimensional scaling (NMDS) based on Bray-Curtis distance at the OTU level illustrating soil fungal community structure. Abbreviations for sampling sites from north to south are as follows: HZ, Huzhong; LS, Liangshui; CB, Changbai; DL, Dongling; TY, Taiyue; SN, Shennong; JL, Jiulian; DH, Dinghu; and JF, Jianfeng.

The NMDS clearly separated the nine forest soil samples. Thus, there were different fungal community structures among the nine forest types (Figure 2). The ANOSIM analyses confirmed significant differences between forest pairs in terms of fungal community structure (*p* < 0.05; Supplementary Table 3). The soil samples from the three low-latitude forests (JL, DH, and JF) were segregated from all others along the first NMDS axis (Figure 2). Further, the three low-latitude forests (JL, DH, and JF) were segregated from each other along the second NMDS axis, and the same was true for the six high-latitude forests (HZ, LS, CB, DL, TY, and SN).

### Association Between Plant Traits and Fungal α-Diversity

Mean annual temperature and precipitation increased from north to south. The soil organic carbon, TP and TN were higher in the temperate forests than the subtropical and tropical forests. There was no obvious change trend in soil C/N. The pH of the low-latitude soils was lower than those of the high-latitude soils (Supplementary Table 1). The CWM of leaf and root C and root N presented no latitudinal trend. In contrast, the leaf N, P and root P levels were relatively lower in the subtropical and tropical forest soils. The CWM root biomass and SRL were relatively lower in the low-latitude forest soils (Supplementary Table 1).

**FIGURE 3:**
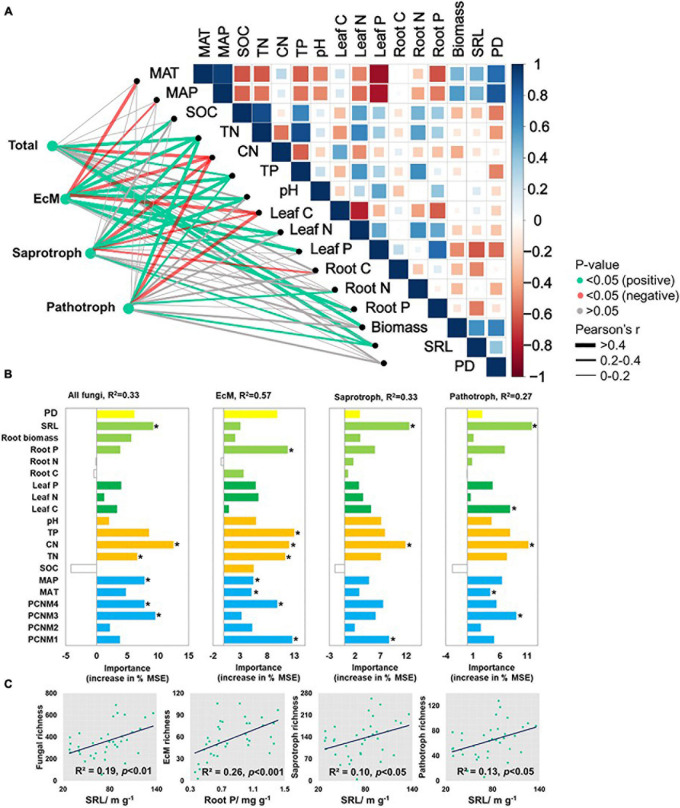
**(A)** Environmental variables correlated with total and major fungal functional richness in nine forest ecosystems. Correlation and significance were determined by Pearson’ tests. **(B)** Random forest analysis identifying best individual predictors of fungal richness including plant traits, soil properties, and climatic and spatial factors. **(C)** Scatterplots showing relationships with most significant drivers. MSE: mean square error. *p < 0.05.

Pearson’s correlations disclosed several significant associations among fungal functional richness and biotic and abiotic environmental factors (Figure 3A). Total fungi and functional fungal guilds were negatively correlated with soil C/N (*p* < 0.05) but positively correlated with TP (*p* < 0.05). SRL (*p* < 0.05) was positively correlated with fungal richness except for EcM fungi. Soil pH (*p* < 0.05) was positively correlated with EcM and saprotrophic fungal richness. Total fungal richness was positively correlated with root biomass (*p* < 0.05). EcM fungal richness was negatively correlated with leaf C, and positively correlated with leaf N and P content (*p* < 0.05). Saprotrophic fungal richness was negatively correlated with plant (including root and leaf) C content, and was positively correlated with leaf N (*p* < 0.05). Pathotrophic fungal richness was positively correlated with root N and negatively correlated with leaf C content (*p* < 0.05). There was no significant correlation between fungal and plant richness.

After all variables were entered into a best ordinary least squares (OLS) multiple regression (Supplementary Table 2), total fungal richness was explained mainly by soil C/N (45.3%) followed by SRL (23.1%), spatial factors (20.4%), SOC (8.7%), and root carbon content (2.6%). EcM fungal richness was determined mainly by soil C/N (51.9%), pH (25.7%), SOC (12.7%), root carbon content (2.2%), and spatial factors (7.5%) (*p* < 0.001). Saprotrophic and pathotrophic fungal richness were affected by C/N, SRL, SOC, and principal coordinates of neighboring matrices (PCNM4) (*p* < 0.001).

**FIGURE 4:**
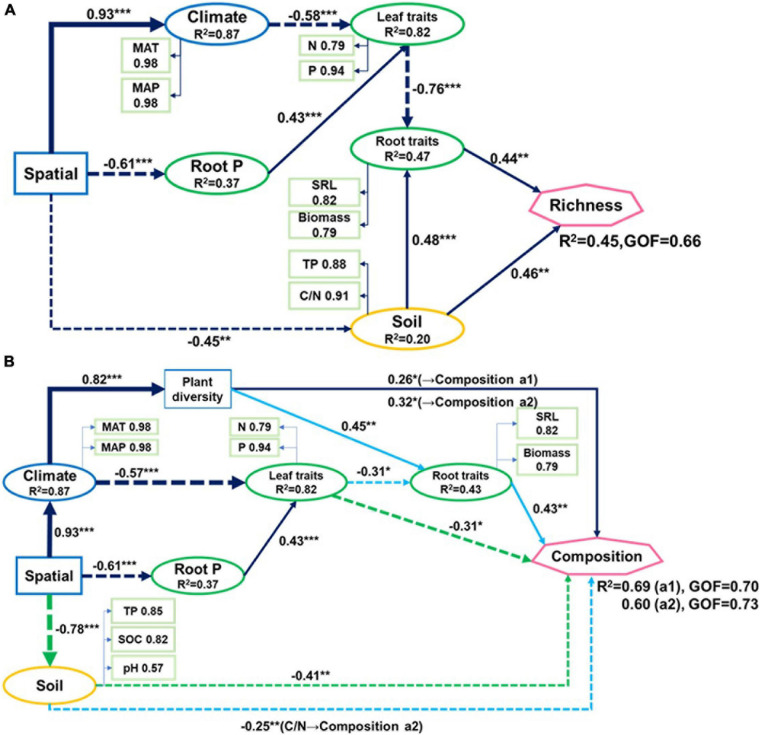
Partial least-squares path models showing **(A)** relationships of fungal richness and **(B)** community composition (two axes from non-metric multidimensional scaling) with climatic and spatial factors, plant traits, and soil properties of nine forest ecosystems. The solid line represents a positive correlation, the dotted line represents a negative correlation. Green line in **(B)** indicates paths to composition a1. Blue line indicates paths to composition a2. Black line indicates paths common to compositions a1 and a2. GOF: goodness-of-fit. Arrow widths are proportional to strengths of causal relationships supplemented by standardized path coefficients (*p < 0.05; **p < 0.01; ***p < 0.001). R^2^ indicate explained variances in response variables.

Random forest tree and PLS-PMs analyses identified the main predictors and the direct and indirect effects of the variables explaining fungal richness (Figures 3B, 4A). SRL, soil C/N, and spatial factors were selected as significant predictors of soil total, saprotrophic, and pathotrophic fungal richness. SRL explained 19.0, 10.0, and 13.0% of the variations in the richness of total fungi, saprotrophs, and pathotrophs, respectively (Figure 3C). Root P, soil TP, C/N, and spatial factors were the major predictors of EcM fungal richness (Figure 3B).

Spatial factors, climate, soil properties, and plant traits explained 45% of the variations in soil fungal richness (Figure 4A). Only soil TP and C/N, root biomass, and SRL directly influenced soil fungal richness. Spatial factors and climate were indirectly associated with fungal richness as they affected plant nutrient concentration which is related to root biomass and SRL. The soil explained most of the fungal richness followed by root traits and leaf N and P.

### Association Between Plant Traits and Fungal Community Composition

**FIGURE 5:**
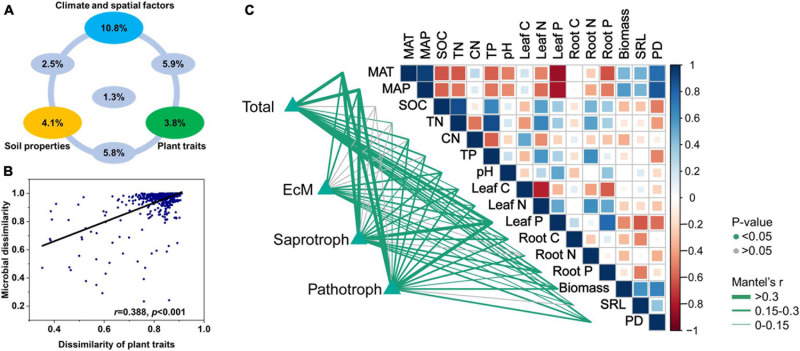
**(A)** Variation partitioning modeling identifying relative contributions of plant traits, climatic and spatial factors, and soil properties as predictors of soil fungal community composition at OTU level. **(B)** Relationship between Bray-Curtis community dissimilarity and Euclidean distance matrix for plant traits. Each data point represents Bray-Curtis dissimilarity score for two samples and Euclidean distance between them. **(C)** Environmental variables correlated with total and major functional fungal community structures in nine forest ecosystems. Correlations and significance were determined by Mantel tests based on 999 permutations.

The dissimilarities among soil fungal communities were significantly correlated with the Euclidean distances among plant traits (Figures 5B,C). Hence, the latter influence fungal community structure. Variation partitioning modeling (Figure 5A) revealed that spatioclimatic and edaphic variables and plant traits explained 20.5, 13.7, and 16.5% of the variation in fungal community composition, respectively. Plant traits alone explained 3.8% of the variation in fungal community composition and contributed to 12.7% of the total variation by interacting with the edaphic and spatioclimatic variables.

**FIGURE 6:**
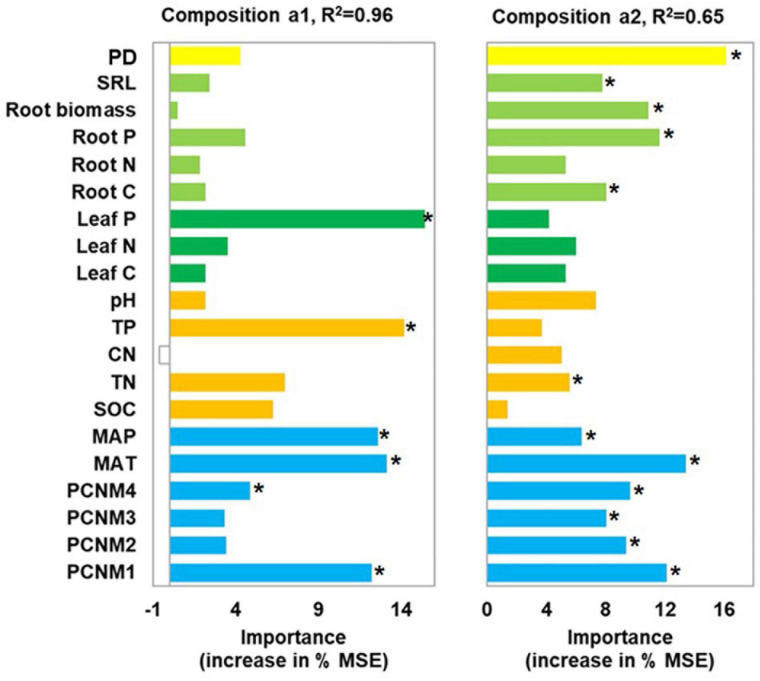
Random forest analysis identifying best individual predictors of fungal community composition in nine forest ecosystems. MSE: mean square error. Community compositions a1 and a2 represent first and second NMDS axes. Compositional variation is represented by Bray-Curtis distance matrix at OTU level. *p < 0.05.

Leaf P content, soil TP, climate, and spatial factors were selected as significant drivers of the first soil fungal community NMDS axis. Climate, spatial factors, and root traits were the predictors of the second NMDS axis (Figure 6). PLS-PMs explained 69 and 60% of the variance in the first and second soil fungal community NMDS axes, respectively (Figure 4B). The root and leaf traits directly affected soil fungal composition. Root biomass allocation had the largest direct effect (composition a2, path coefficient = 0.43). Leaf traits indirectly affected fungal composition (composition a2) by altering root traits (Figure 4B), besides, leaf traits had direct effects on fungal composition (composition a1). Soil TP, SOC, and pH had the greatest direct effects of all variables on soil fungal community composition (composition a1, path coefficient = −0.41).

## Discussion

**FIGURE 7:**
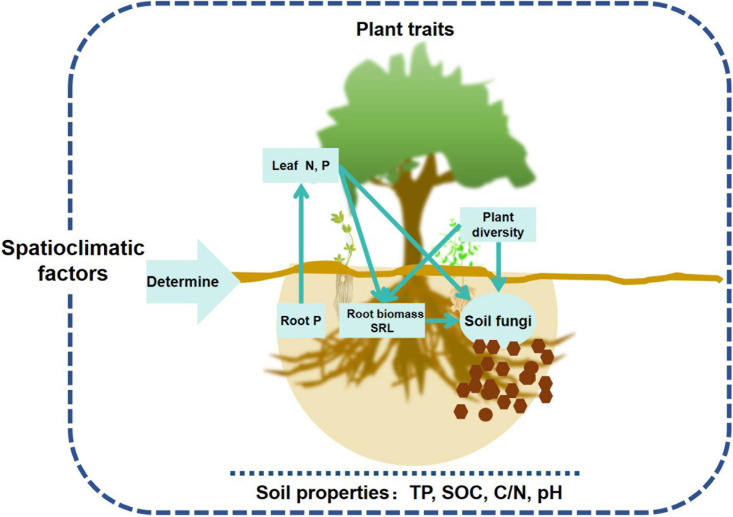
Conceptual framework of aboveground and belowground plant traits’ effects on topsoil fungal communities in forest ecosystems across a wide range of latitudes.

Plants are assumed to play an important role in structuring soil microbial communities, but most studies have only used aboveground plant community instead in exploring the relationships between microbes and plants at the community level (López-Angulo et al., 2020). This study comprehensively evaluated the effects of above and belowground plant traits on soil fungal community, and demonstrated that aboveground leaf traits (nutrients) and belowground root traits (biomass, SRL, and nutrients) largely influenced topsoil fungal richness and community composition in forest ecosystems (Figure 7). Spatial and climatic factors exerted indirect effects on soil fungal community mainly via affecting soil properties, nutrient and energy exchange between roots and leaves (Figures 4, 7).

In contrast to global-scale soil fungal biogeographic patterns (Tedersoo et al., 2014; Bahram et al., 2018), there was no trend in the richness of overall soil fungi, major taxonomic and functional fungal groups with latitude along the nine forest ecosystems studied here besides EcM. EcM fungal richness increased with latitude and reached maxima in temperate forests (Supplementary Figure 4). Most EcM fungi belong to stress-tolerant taxonomic groups (Beck et al., 2013; Treseder et al., 2014). Moreover, the number of colonizable roots increases with the number of EcM plants which, in turn, furnish carbon for EcM fungi in temperate forests (Tedersoo et al., 2012; Tedersoo et al., 2014). In this type of ecosystems, EcM play a dominant role in N and P acquisition from the soil and transfer to host plant roots (Tedersoo et al., 2012).

Various climatic and soil factors may affect soil fungal richness and community composition. Of these, temperature and precipitation are the most important on a large scale (Tedersoo et al., 2014; Zhou et al., 2016; Bahram et al., 2018). Climatic conditions directly determine fungal species survival and soil colonization. They also affect soil fungal communities by indirectly influencing local vegetation. Soil C/N and pH also predict topsoil fungal richness and community composition at the global scale (Tedersoo et al., 2014; Bahram et al., 2018). Fungal distribution is limited by resource availability and soil C/N strongly influences soil fungal richness (Tedersoo et al., 2014). In contrast, the soil fungal community was relatively less responsive to pH than the soil bacterial community (Tedersoo et al., 2014; Bahram et al., 2018).

Plant inputs to the soil determine soil microbial C requirements (Cline et al., 2018). Previous studies showed that SRL is positively correlated with root exudation (Guyonnet et al., 2018) and negatively correlated with fine root lifespan (Luke McCormack et al., 2012). The positive association between SRL and soil fungal richness observed here was consistent with the fact that plant traits regulate the amount of easily metabolizable substrate available to the soil fungal community. Root litter and exudates are more readily metabolized than aboveground litter by soil microbes (Cotrufo et al., 2013). However, in this study, leaf traits only indirectly influenced fungal richness by allocating photosynthate to the roots. Moreover, plant richness was not related to fungal richness. A recent study (Delgado-Baquerizo et al., 2018) underscored the importance of aboveground plant community traits such as diversity, cover, and leaf traits in the prediction of soil microbial community diversity. Only plant cover had a negative effect on soil fungal diversity and the associations between plant species and soil fungal richness varied with sampling site (Delgado-Baquerizo et al., 2018). The outcome of the present study corroborated the preceding results. Here, we showed that belowground plant traits accounted for most of the variation in soil fungal richness. Taken together, these findings suggest that soil fungal richness is driven (i) directly by root traits as the roots furnish resources via exudates and decomposition, and (ii) indirectly by leaf traits as the leaves allocate photosynthate to the roots. Besides nutrient traits, specific leaf area and dry matter content were related to soil fungal community (de Vries et al., 2012; Pei et al., 2016; De Long et al., 2019). The limitation of this study is that leaf traits only include three nutrient traits; however, root traits include extra morphological and biomass. This may cause bias in the assessment of the relative importance of leaf and root traits in affecting soil fugal communities.

Since different fungal groups have different plant C sources, are they related to different plant traits? We found significant correlations between SRL and richness of saprotrophic and pathotrophic fungi (Figure 3A). This observation is consistent with a resource strategy based on plant material metabolism (Goldmann et al., 2015; Schimann et al., 2017). Saprotrophic fungi derive their energy from plant litter decay and soil organic matter. Pathotrophic fungi rely mainly on living host plants for their metabolic energy. Nevertheless, there were significant correlations among root P, leaf N, and EcM fungal richness. This finding is consistent with a resource strategy that depends upon plant photoassimilates. EcM fungi utilize photosynthate in fine roots and provide the host plant with N and P. Host plants can increase their C allocation to roots in order to acquire more P (Kou et al., 2018).

Spatial distributions of soil fungi are the results of the combined effects of fungal diversification, climate, soil geography, and plant community selection (Tedersoo et al., 2014). In the present study, most of the variation in the soil fungal communities in the nine forest ecosystems was the result of relative differences in their spatial and climatic factors. The soil fungal community compositions differed between the evergreen broad-leaved forest (JL, DH, and JF) and others (HZ, LS, CB, DL, TY, and SN) primarily because of the dramatic differences between these two ecosystems in terms of their climate and soil properties. Spatial and climatic factors influence soil fungal communities mainly by indirectly altering soil properties and plant traits. Plant species richness and leaf and root traits directly and jointly affect soil fungal community compositions (Figure 7). These results are consistent with those of previous reports. Plant diversity (He et al., 2017) and leaf N affect fungal community composition (Delgado-Baquerizo et al., 2018). Furthermore, we demonstrated that both aboveground and belowground plant traits contributed to variation in soil fungal community composition as they determine the amounts of plant resources entering the soil. In accordance with Legay et al. (2014), the present study showed that it is the root traits rather than leaf traits that drive soil fungal community structure. Roots directly impact easily metabolizable C resources (root decomposition and exudates) (de Vries et al., 2012; Grigulis et al., 2013; Delgado-Baquerizo et al., 2018).

Soil fungi are vital links in the food web and energy channels. Soil fungal richness and community composition are mediated by fungal predators, competitors, and prey (de Vries et al., 2013). Microbial consumers and bioturbators such as protozoa, nematodes, and earthworms influence soil fungi by mineralizing soil nutrients and augmenting soil nutrient bioavailability (de Vries et al., 2013). Nevertheless, the aforementioned processes and interactions have not been fully elucidated and merit further investigation.

## Conclusion

Here, we detected no obvious latitudinal trend in soil fungal richness. Nevertheless, the present study demonstrated that the soil fungal community composition of low-latitude evergreen forests markedly differed from those of high-latitude deciduous and coniferous forests. Abiotic and biotic factors collectively explained 45% and > 60% of the soil fungal richness and community composition, respectively. Root biomass and specific root length exerted the strongest direct effects on soil fungal richness and community composition at the community level. Leaf nitrogen and phosphorus substantially affected richness mainly via their influences on root biomass allocation, while they exerted both direct and indirect effects on community composition. Moreover, spatial and climatic factors mainly affected soil and plant properties which, in turn, influenced soil fungal community composition. The results of this study demonstrated that plant traits predict soil fungal community distribution at the regional scale. Hence, plant traits could improve the accuracy and reliability of predicting of soil fungal communities in forest ecosystems across a wide range of latitudes.

## Data Availability Statement

The original contributions presented in the study are publicly available. This data can be found here: NCBI repository, accession number PRJNA717126.

## Author Contributions

JTi and GY designed the experiment. JTe and JTi collected and analyzed the data. JTe drafted the manuscript with help of JTi, GY, RB, YK, and JZ. All authors gave final approval for publication.

## Conflict of Interest

The authors declare that the research was conducted in the absence of any commercial or financial relationships that could be construed as a potential conflict of interest.

## Supplementary Material

Supplementary Figure 1 Distribution of typical forest ecosystems along the North-South Transect of eastern China (NSTEC). The abbreviations of sampling sites from north to south are as follows: HZ, Huzhong; LS, Liangshui; CB, Changbai; DL, Dongling; TY, Taiyue; SN, Shennong; JL, Jiulian; DH, Dinghu; JF, Jianfeng.

Supplementary Figure 2 Relative abundances of the dominant fungal classes.

Supplementary Figure 3 Relationships between relative abundance (%) and latitude for dominant fungal phyla.

Supplementary Figure 4 Relationships between richness and relative abundance with latitude for fungal functional guild.

Supplementary Figure 5 A priori path model for plant traits explains latitudinal patterns in topsoil fungal communities. Arrows indicate causal directed relationships between variables. The climate (1), soil (2), plant diversity (4) (Harrison et al., 2020), and plant traits (5) (7) (8) (Wang et al., 2018; Zhang et al., 2018) vary with latitude across wide spatial scales. These biotic and abiotic factors influence soil fungal community, including geographic isolation (6) (Cox et al., 2016), climate (10) (Tedersoo et al., 2014; Zhou et al., 2016), soil properties (15) (Leifheit et al., 2014; Guo et al., 2019), and plant diversity (18) (Peay et al., 2013; Cline et al., 2018). Plant diversity influences the quantity and composition of plant inputs (19) (20) (21) (Wardle et al., 2004; Bardgett and van der Putten, 2014). CWM of leaf traits such as specific area, dry matter content, and nitrogen concentration can impact soil fungal community by influencing litter decomposition (25) (Quested et al., 2007; Fortunel et al., 2009; Eichenberg et al., 2015), soil nutrients cycles, and belowground processes (de Vries et al., 2012; Pei et al., 2016; De Long et al., 2019). Fine root decomposition and root exudates are major C contributors to forest soil microbial communities and regulate microbial metabolism (Freschet and Cornelissen, 2013; Ponge, 2013). The chemical traits of fine roots are the best indicators of community-level root decomposition and root exudates (24) (Prieto et al., 2016; See et al., 2019). Fine roots with large specific root lengths (SRL) are positively correlated with fungal: bacterial (F: B) ratios (27) (Legay et al., 2014). In addition, plant diversity (3) (4) (13) (Harrison et al., 2020) and leaf nutrients (5) (9) (14) (Zhang et al., 2018), root nutrients (8) (12) (17) (Zhang et al., 2018) and root biomass allocation (7) (11) (16) are all strongly depend upon climatic and edaphic variables. Leaf and root traits often show coordinated variation in stoichiometry (22) (Freschet and Cornelissen, 2013). Leaf and root nutrients, scale with plant photosynthetic and relative growth rates (23) (26) (Reich, 2014).
