# Supplementary material for: Aboveground and Belowground Plant Traits Explain Latitudinal Patterns in Topsoil Fungal Communities From Tropical to Cold Temperate Forests
Source: Front Microbiol. 2021 Jun 10;12:633751. doi: 10.3389/fmicb.2021.633751 (PMC8222577; doi:10.3389/fmicb.2021.633751)
Supplement: Supplementary Figure 1 — Distribution of typical forest ecosystems along the North-South Transect of eastern China (NSTEC). The abbreviations of sampling sites from north to south are as follows: HZ, Huzhong; LS, Liangshui; CB, Changbai; DL, Dongling; TY, Taiyue; SN, Shennong; JL, Jiulian; DH, Dinghu; JF, Jianfeng. [file Data_Sheet_1.docx]

Supplementary Material

# Supplementary Data

Supplementary Material should be uploaded separately on submission. Please include any supplementary data, figures and/or tables. All supplementary files are deposited to FigShare for permanent storage and receive a DOI.

Supplementary material is not typeset so please ensure that all information is clearly presented, the appropriate caption is included in the file and not in the manuscript, and that the style conforms to the rest of the article. To avoid discrepancies between the published article and the supplementary material, please do not add the title, author list, affiliations or correspondence in the supplementary files.

# Supplementary Figures and Tables

## Supplementary Figures


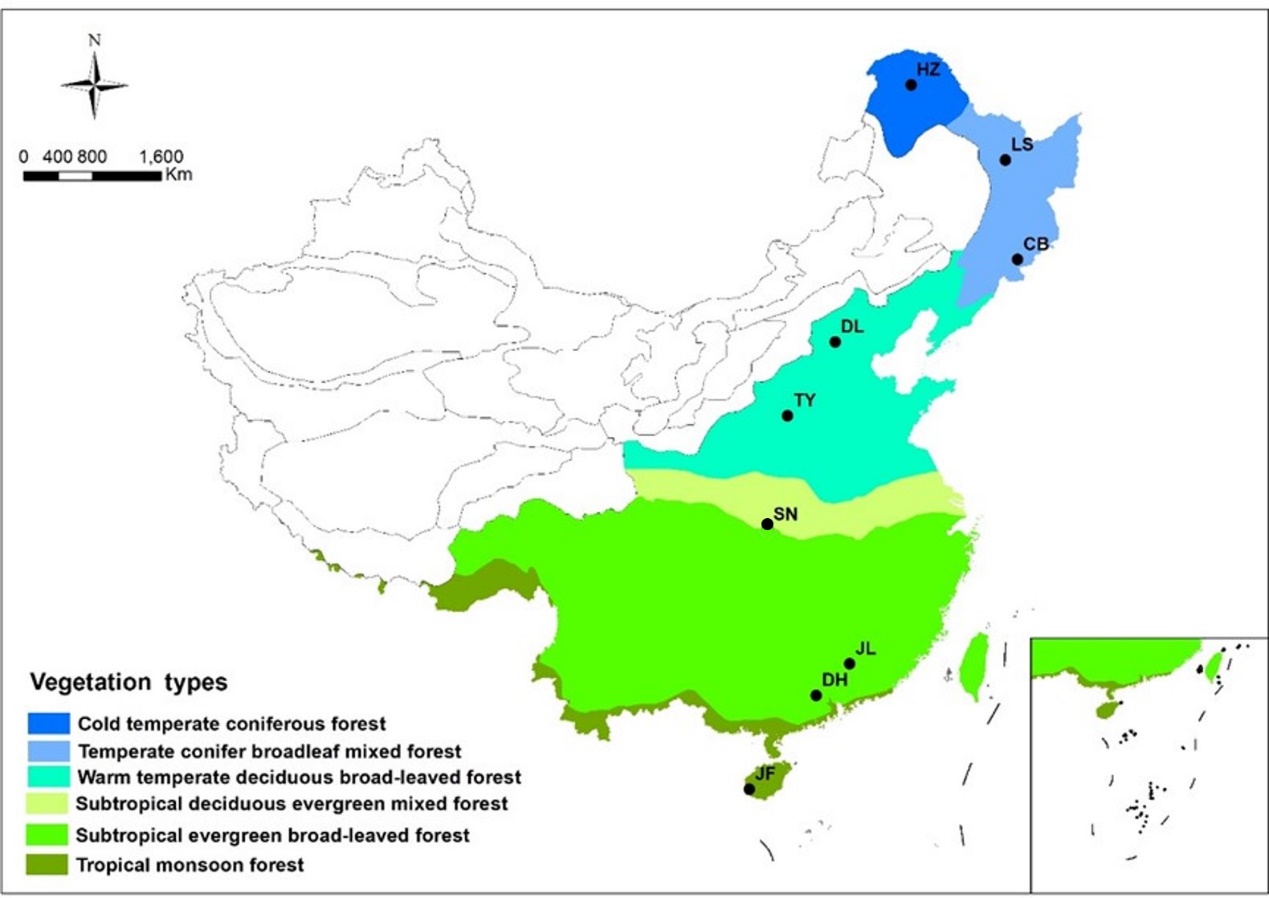


**Supplementary Figure 1.** Distribution of typical forest ecosystems along the North-South Transect of eastern China (NSTEC). The abbreviations of sampling sites from north to south are as follows: HZ, Huzhong; LS, Liangshui; CB, Changbai; DL, Dongling; TY, Taiyue; SN, Shennong; JL, Jiulian; DH, Dinghu; JF, Jianfeng.

**
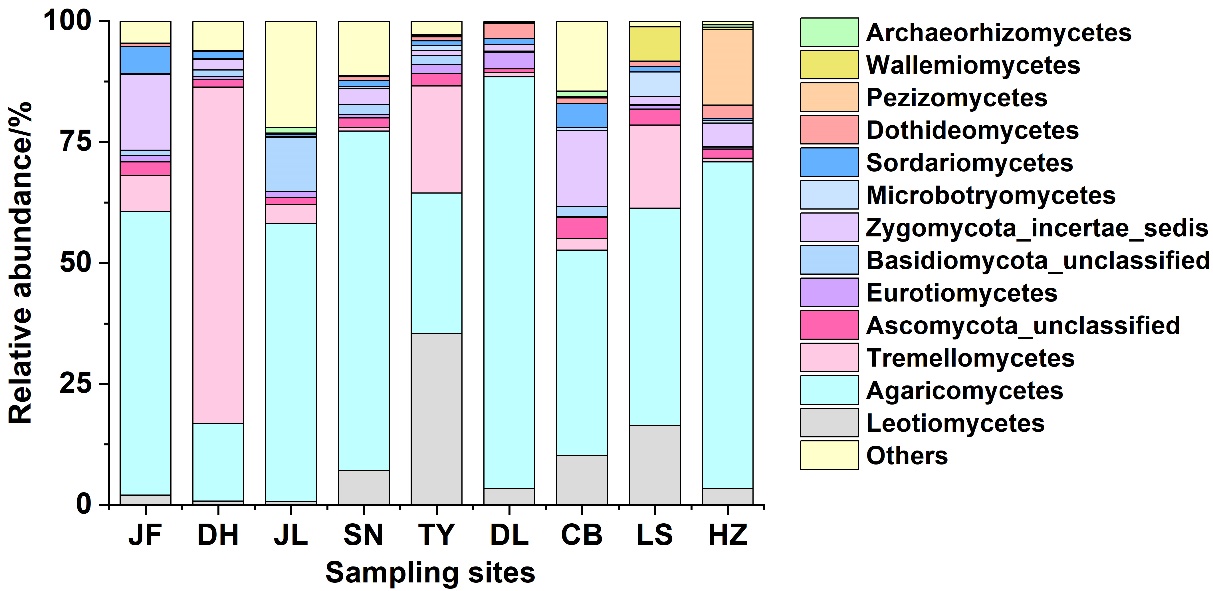
**

**Supplementary Figure 2.** Relative abundances of the dominant fungal classes.

**
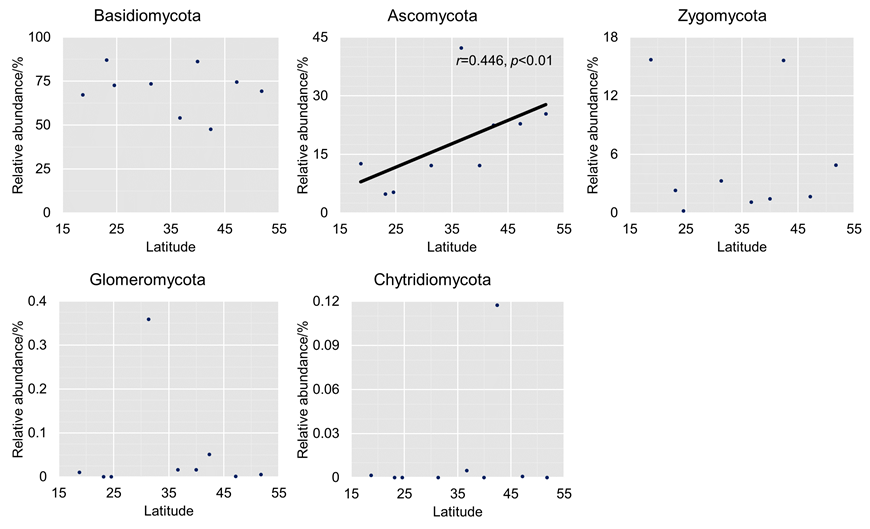
**

**Supplementary Figure 3.** Relationships between relative abundance (%) and latitude for dominant fungal phyla.


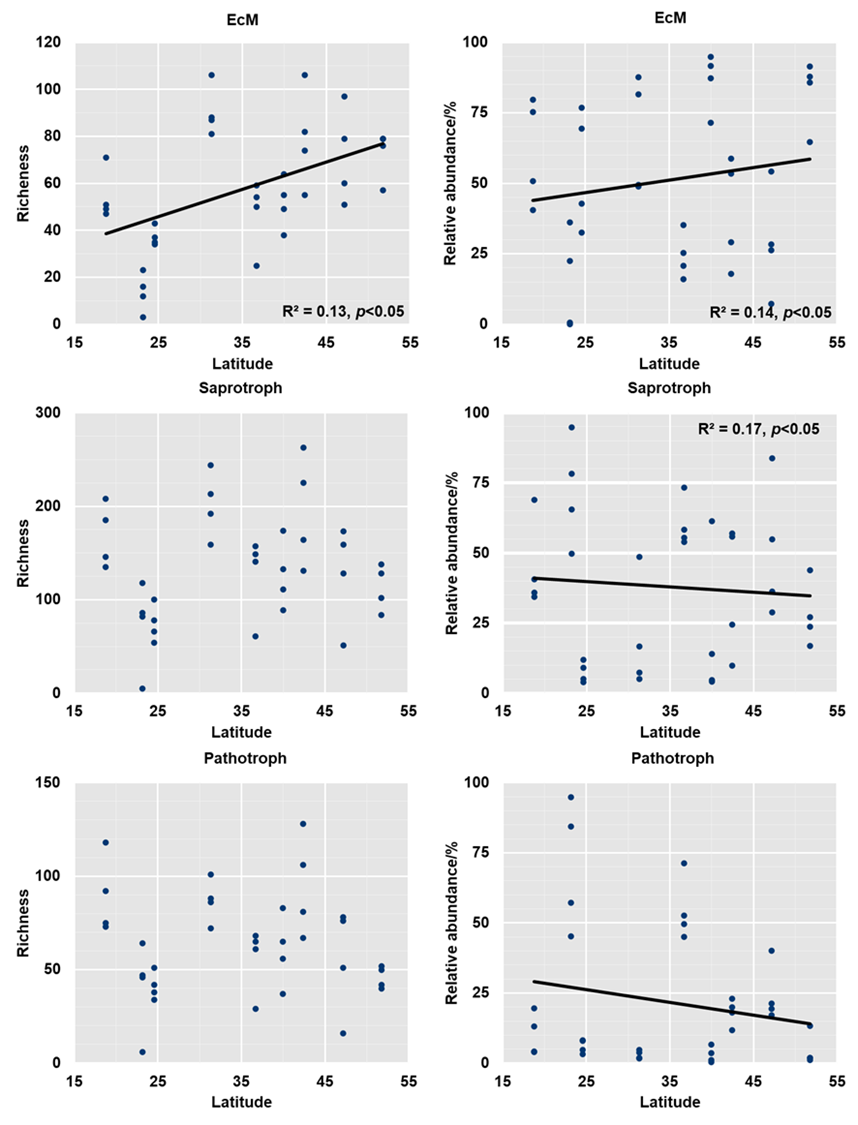


**Supplementary Figure 4.** Relationships between richness and relative abundance with latitude for fungal functional guilds.


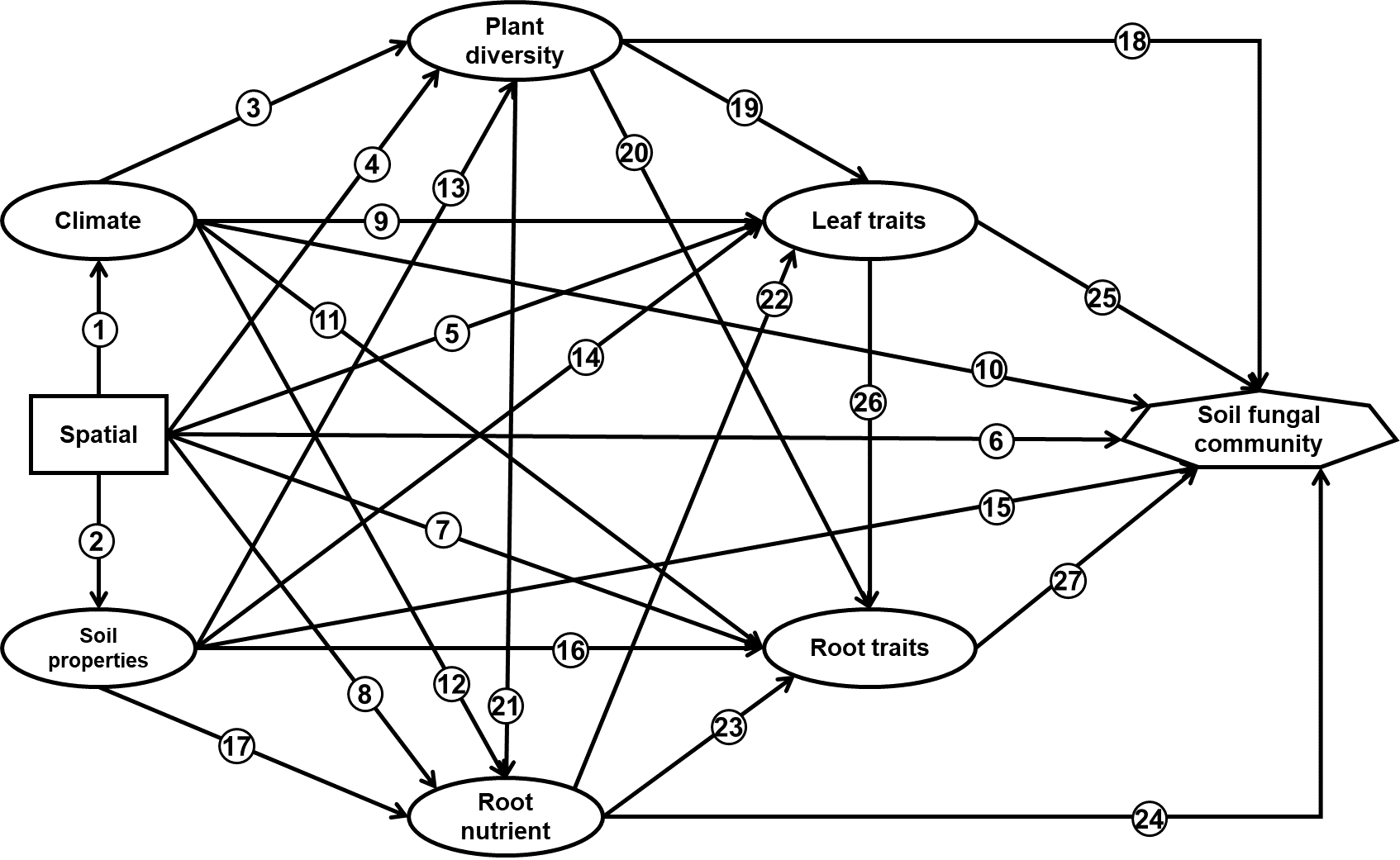


**Supplementary Figure 5.** A priori path model for plant traits explains latitudinal patterns in topsoil fungal communities. Arrows indicate causal directed relationships between variables. The climate (1), soil (2), plant diversity (4) (Harrison et al., 2020), and plant traits (5) (7) (8) (Wang et al., 2018; Zhang et al., 2018) vary with latitude across wide spatial scales. These biotic and abiotic factors influence soil fungal community, including geographic isolation (6) (Cox et al., 2016), climate (10) (Tedersoo et al., 2014; Zhou et al., 2016), soil properties (15) (Leifheit et al., 2014; Guo et al., 2019), and plant diversity (18) (Peay et al., 2013; Cline et al., 2018). Plant diversity influences the quantity and composition of plant inputs (19) (20) (21) (Wardle et al., 2004; Bardgett and van der Putten, 2014). Community-weighted means of leaf traits such as specific area, dry matter content, and nitrogen concentration can impact soil fungal community by influencing litter decomposition (25) (Quested et al., 2007; Fortunel et al., 2009; Eichenberg et al., 2015), soil nutrients cycles, and belowground processes (de Vries et al., 2012; Pei et al., 2016; De Long et al., 2019). Fine root decomposition and root exudates are major C contributors to forest soil microbial communities and regulate microbial metabolism (Freschet and Cornelissen, 2013; Ponge, 2013). The chemical traits of fine roots are the best indicators of community-level root decomposition and root exudates (24) (Prieto et al., 2016; See et al., 2019). Fine roots with large specific root lengths (SRL) are positively correlated with fungal: bacterial (F: B) ratios (27) (Legay et al., 2014). In addition, plant diversity (3) (4) (13) (Harrison et al., 2020) and leaf nutrients (5) (9) (14) (Zhang et al., 2018), root nutrients (8) (12) (17) (Zhang et al., 2018) and root biomass allocation (7) (11) (16) are all strongly depend upon climatic and edaphic variables. Leaf and root traits often show coordinated variation in stoichiometry (22) (Freschet and Cornelissen, 2013). Leaf and root nutrients, scale with plant photosynthetic and relative growth rates (23) (26) (Reich, 2014).

## Supplementary Tables

**Supplementary Table 1.** Summary statistics of the geographic and environmental characteristics of the nine forest sites.

|  |  | Latitude  (°N) | Longitude (°E) | MAT  (℃) | MAP  (mm) | SOC  (g/kg) | TN  (g/kg) | C/N | TP  (g/kg) | pH | Leaf traits | | | Root traits | | | | | Plant diversity |
| --- | --- | --- | --- | --- | --- | --- | --- | --- | --- | --- | --- | --- | --- | --- | --- | --- | --- | --- | --- |
|  |  |  |  |  |  |  |  |  |  |  | C (mg/g) | N (mg/g) | P (mg/g) | C  (mg/g) | N  (mg/g) | P  (mg/g) | Biomass  (t/hm2) | SRL (m/g) |  |
| HZ | Mean | 51.8 | 123.0 | -3.7 | 473.0 | 42.2 | 3.1 | 13.8 | 0.8 | 6.8 | 488.0 | 19.7 | 2.1 | 502.4 | 8.6 | 1.1 | 14.1 | 35.0 | 22 |
|  | SD |  |  |  |  | 3.3 | 0.7 | 2.9 | 0.1 | 0.2 | 0.3 | 0.1 | 0.0 | 2.3 | 0.6 | 0.1 | 7.0 | 6.6 | 7 |
| LS | Mean | 47.2 | 128.9 | 0.0 | 648.3 | 68.9 | 4.6 | 14.9 | 0.6 | 6.1 | 474.9 | 22.7 | 1.6 | 490.3 | 9.3 | 1.4 | 29.5 | 36.5 | 40 |
|  | SD |  |  |  |  | 22.6 | 1.2 | 2.2 | 0.1 | 0.3 | 3.5 | 1.1 | 0.1 | 3.0 | 0.6 | 0.0 | 3.3 | 9.7 | 1 |
| CB | Mean | 42.4 | 128.1 | 2.8 | 691.0 | 70.0 | 6.4 | 11.1 | 1.8 | 6.3 | 473.1 | 25.8 | 1.2 | 484.1 | 15.0 | 0.7 | 29.3 | 82.2 | 27 |
|  | SD |  |  |  |  | 7.3 | 0.9 | 0.9 | 0.4 | 0.3 | 1.7 | 0.8 | 0.0 | 1.0 | 0.1 | 0.0 | 2.6 | 4.7 | 3 |
| DL | Mean | 40.0 | 115.4 | 6.6 | 539.1 | 39.5 | 3.1 | 12.7 | 0.6 | 6.9 | 470.1 | 25.7 | 1.7 | 489.8 | 11.3 | 1.1 | 32.4 | 64.1 | 49 |
|  | SD |  |  |  |  | 1.9 | 0.2 | 0.4 | 0.0 | 0.1 | 11.3 | 1.7 | 0.2 | 10.0 | 0.8 | 0.1 | 4.2 | 20.3 | 9 |
| TY | Mean | 36.7 | 112.1 | 6.0 | 644.4 | 43.4 | 2.6 | 17.0 | 0.5 | 6.8 | 496.2 | 18.3 | 1.1 | 471.6 | 10.9 | 0.5 | 30.7 | 92.0 | 23 |
|  | SD |  |  |  |  | 7.7 | 0.4 | 0.7 | 0.0 | 0.2 | 7.6 | 2.6 | 0.1 | 6.3 | 0.9 | 0.0 | 6.7 | 6.5 | 9 |
| SN | Mean | 31.3 | 110.5 | 8.5 | 1446.7 | 38.3 | 3.8 | 10.1 | 0.8 | 6.9 | 479.4 | 20.3 | 1.2 | 474.4 | 7.8 | 1.0 | 79.8 | 115.5 | 77 |
|  | SD |  |  |  |  | 6.2 | 0.3 | 0.8 | 0.0 | 0.1 | 13.0 | 1.3 | 0.1 | 7.8 | 2.4 | 0.2 | 6.0 | 15.7 | 10 |
| JL | Mean | 24.6 | 114.4 | 18.2 | 1769.9 | 33.8 | 2.3 | 14.3 | 0.4 | 5.8 | 486.8 | 18.0 | 0.8 | 488.4 | 8.9 | 0.6 | 58.4 | 70.4 | 85 |
|  | SD |  |  |  |  | 6.8 | 0.3 | 1.4 | 0.0 | 0.7 | 4.8 | 0.3 | 0.0 | 1.9 | 2.0 | 0.1 | 8.1 | 21.1 | 2 |
| DH | Mean | 23.2 | 112.5 | 21.8 | 1927.0 | 29.4 | 1.8 | 16.7 | 0.2 | 5.4 | 498.2 | 17.6 | 0.7 | 495.3 | 8.9 | 0.4 | 32.3 | 83.9 | 53 |
|  | SD |  |  |  |  | 2.6 | 0.2 | 0.9 | 0.0 | 0.3 | 5.3 | 0.5 | 0.1 | 2.4 | 1.0 | 0.0 | 5.3 | 21.9 | 10 |
| JF | Mean | 18.7 | 108.9 | 23.2 | 2265.8 | 28.4 | 1.9 | 14.6 | 0.2 | 6.3 | 475.5 | 19.1 | 0.8 | 489.9 | 9.4 | 0.7 | 51.3 | 94.9 | 116 |
|  | SD |  |  |  |  | 3.2 | 0.1 | 1.6 | 0.0 | 0.1 | 3.3 | 0.5 | 0.0 | 10.1 | 0.7 | 0.1 | 20.3 | 23.3 | 10 |

MAP, mean annual precipitation; MAT, mean annual temperature; SOC, soil total carbon; TN, soil total nitrogen; C/N, soil carbon, nitrogen ratios; TP, soil total phosphorus; Leaf/Root C, leaf/root carbon content; Leaf/Root N, leaf/root nitrogen content; Leaf/Root P, leaf/root phosphorus content; SRL, specific root length.

**Supplementary Table 2.** Summary of the best ordinary least squares (OLS) multiple regression models of the species richness of different functional fungi with environmental variables.

|  | Variables retained in the model and its contribution (%) | R^2^_adj_ | *p* value |
| --- | --- | --- | --- |
| Total fungi | C/N (45.3), SRL (23.1), PCNM2 (12.5), SOC (8.7), PCNM4 (7.9), Root C (2.6) | 0.577 | <0.001 |
| EcM | C/N (51.9), pH (25.7), SOC (12.7), PCNM2 (7.5),  Root C (2.2) | 0.680 | <0.001 |
| Saprotroph | C/N (55.0), SRL (22.8), SOC (13.5), PCNM4 (8.6) | 0.516 | <0.001 |
| Pathotroph | C/N (52.4), SRL (16.7), PCNM4 (14.4), SOC (16.6) | 0.523 | <0.001 |

C/N, soil carbon/nitrogen ratios; SRL, specific root length; PCNM2/4, spatial factors; SOC, soil total carbon; TP, soil total phosphorus; Root C, leaf/root carbon content.

**Supplementary Table 3.** ANOSIM F statistic and associated p value of fungal community structure between pairwise forest ecosystems

|  | HZ | LS | CB | DL | TY | SN | JL | DH |
| --- | --- | --- | --- | --- | --- | --- | --- | --- |
| LS | 4.42  (0.030) |  |  |  |  |  |  |  |
| CB | 4.44  (0.032) | 3.25  (0.031) |  |  |  |  |  |  |
| DL | 3.12  (0.023) | 2.66  (0.022) | 2.57  (0.024) |  |  |  |  |  |
| TY | 5.45  (0.027) | 2.63  (0.031) | 4.42  (0.033) | 3.00  (0.030) |  |  |  |  |
| SN | 4.41  (0.032) | 3.70  (0.031) | 3.44  (0.032) | 2.29  (0.034) | 4.24  (0.028) |  |  |  |
| JL | 3.27  (0.045) | 2.59  (0.024) | 2.75  (0.024) | 1.83  (0.030) | 3.20  (0.032) | 2.55  (0.037) |  |  |
| DH | 9.13  (0.021) | 6.34  (0.035) | 7.47  (0.028) | 4.63  (0.027) | 9.02  (0.030) | 6.81  (0.036) | 4.25  (0.025) |  |
| JF | 6.38  (0.030) | 5.06  (0.022) | 4.30  (0.037) | 3.34  (0.024) | 6.39  (0.032) | 4.79  (0.038) | 3.20  (0.032) | 8.51  (0.033) |

**Reference**

Bardgett, R.D., and van der Putten, W.H. (2014). Belowground biodiversity and ecosystem functioning. *Nature* 515: 505-511. doi: 10.1038/nature13855.

Cline, L.C., Hobbie, S.E., Madritch, M.D., Buyarski, C.R., Tilman, D., and Cavender-Bares, J.M. (2018). Resource availability underlies the plant-fungal diversity relationship in a grassland ecosystem. *Ecology* 99: 204-216. doi: 10.1002/ecy.2075.

Cox, F., Newsham, K.K., Bol, R., Dungait, J.A.J., and Robinson, C.H. (2016). Not poles apart: Antarctic soil fungal communities show similarities to those of the distant Arctic. *Ecology Letters* 19: 528-536. doi: 10.1111/ele.12587.

De Long, J.R., Jackson, B.G., Wilkinson, A., Pritchard, W.J., Oakley, S., Mason, K.E. et al. (2019). Relationships between plant traits, soil properties and carbon fluxes differ between monocultures and mixed communities in temperate grassland. *Journal of Ecology* 107: 1704-1719. doi: 10.1111/1365-2745.13160.

de Vries, F.T., Manning, P., Tallowin, J.R., Mortimer, S.R., Pilgrim, E.S., Harrison, K.A. et al. (2012). Abiotic drivers and plant traits explain landscape-scale patterns in soil microbial communities. *Ecology Letters* 15: 1230-1239

Eichenberg, D., Trogisch, S., Huang, Y.Y., He, J.S., and Bruelheide, H. (2015). Shifts in community leaf functional traits are related to litter decomposition along a secondary forest succession series in subtropical China. *Journal of Plant Ecology* 8: 401-410

Fortunel, C., Garnier, E., Joffre, R., Kazakou, E., Quested, H., Grigulis, K. et al. (2009). Leaf traits capture the effects of land use changes and climate on litter decomposability of grasslands across Europe. *Ecology* 90: 598-611. doi: Doi 10.1890/08-0418.1.

Freschet, G.T., and Cornelissen, J.H.C. (2013). Linking litter decomposition of above- and below-ground organs to plant-soil feedbacks worldwide. *Journal of Ecology* 101: 943-952

Guo, J., Ling, N., Chen, Z., Xue, C., Li, L., Liu, L. et al. (2019). Soil fungal assemblage complexity is dependent on soil fertility and dominated by deterministic processes. *New Phytologist* 226: 232-243. doi: 10.1111/nph.16345.

Harrison, S., Spasojevic, M.J., and Li, D. (2020). Climate and plant community diversity in space and time. *Proceedings of the National Academy of Sciences* 117: 4464-4470. doi: 10.1073/pnas.1921724117.

Legay, N., Baxendale, C., Grigulis, K., Krainer, U., Kastl, E., Schloter, M. et al. (2014). Contribution of above- and below-ground plant traits to the structure and function of grassland soil microbial communities. *Annals of Botany* 114: 1011-1021. doi: 10.1093/aob/mcu169.

Leifheit, E.F., Veresoglou, S.D., Lehmann, A., Morris, E.K., and Rillig, M.C. (2014). Multiple factors influence the role of arbuscular mycorrhizal fungi in soil aggregation—a meta-analysis. *Plant and Soil* 374: 523-537. doi: 10.1007/s11104-013-1899-2.

Peay, K.G., Baraloto, C., and Fine, P.V.A. (2013). Strong coupling of plant and fungal community structure across western Amazonian rainforests. *Isme Journal* 7: 1852-1861. doi: 10.1038/ismej.2013.66.

Pei, Z.Q., Eichenberg, D., Bruelheide, H., Krober, W., Kuhn, P., Li, Y. et al. (2016). Soil and tree species traits both shape soil microbial communities during early growth of Chinese subtropical forests. *Soil Biology & Biochemistry* 96: 180-190. doi: 10.1016/j.soilbio.2016.02.004.

Ponge, J.-F. (2013). Plant–soil feedbacks mediated by humus forms: A review. *Soil Biology and Biochemistry* 57: 1048-1060. doi: <https://doi.org/10.1016/j.soilbio.2012.07.019>.

Prieto, I., Stokes, A., and Roumet, C. (2016). Root functional parameters predict fine root decomposability at the community level. *Journal of Ecology* 104: 725-733. doi: 10.1111/1365-2745.12537.

Quested, H., Eriksson, O., Fortunel, C., and Garnier, E. (2007). Plant traits relate to whole-community litter quality and decomposition following land use change. *Functional Ecology* 21: 1016-1026. doi: 10.1111/j.1365-2435.2007.01324.x.

Reich, P.B. (2014). The world-wide 'fast-slow' plant economics spectrum: a traits manifesto. *Journal of Ecology* 102: 275-301. doi: Doi 10.1111/1365-2745.12211.

See, C.R., Luke McCormack, M., Hobbie, S.E., Flores-Moreno, H., Silver, W.L., and Kennedy, P.G. (2019). Global patterns in fine root decomposition: climate, chemistry, mycorrhizal association and woodiness. *Ecology Letters* 22: 946-953. doi: 10.1111/ele.13248.

Tedersoo, L., Bahram, M., Polme, S., Koljalg, U., Yorou, N.S., Wijesundera, R. et al. (2014). Global diversity and geography of soil fungi. *Science* 346: 1256688. doi: 10.1126/science.1256688.

Wang, R.L., Wang, Q.F., Zhao, N., Xu, Z.W., Zhu, X.J., Jiao, C.C. et al. (2018). Different phylogenetic and environmental controls of first-order root morphological and nutrient traits: Evidence ofmultidimensional root traits. *Functional Ecology* 32: 29-39. doi: 10.1111/1365-2435.12983.

Wardle, D.A., Bardgett, R.D., Klironomos, J.N., Setala, H., van der Putten, W.H., and Wall, D.H. (2004). Ecological linkages between aboveground and belowground biota. *Science* 304: 1629-1633. doi: DOI 10.1126/science.1094875.

Zhang, J., Zhao, N., Liu, C., Yang, H., Li, M., Yu, G. et al. (2018). C:N:P stoichiometry in China's forests: From organs to ecosystems. *Functional Ecology* 32: 50-60. doi: <https://doi.org/10.1111/1365-2435.12979>.

Zhou, J., Deng, Y., Shen, L., Wen, C., Yan, Q., Ning, D. et al. (2016). Temperature mediates continental-scale diversity of microbes in forest soils. *Nature Communications* 7: 12083. doi: 10.1038/ncomms12083.
